# “Keep That in Mind!” The Role of Positive Affect in Working Memory for Maintaining Goal-Relevant Information

**DOI:** 10.3389/fpsyg.2018.01228

**Published:** 2018-07-19

**Authors:** Jessica S. B. Figueira, Luiza B. Pacheco, Isabela Lobo, Eliane Volchan, Mirtes G. Pereira, Leticia de Oliveira, Isabel A. David

**Affiliations:** ^1^Laboratory of Behavioral Neurophysiology, Physiology and Pharmacology Department, Biomedical Institute, Universidade Federal Fluminense, Niteroi, Brazil; ^2^Group of Psychobiology, Nucleo em Ecologia e Desenvolvimento Socio-Ambiental de Macae, Universidade Federal do Rio de Janeiro, Macae, Brazil; ^3^Laboratory of Neurobiology II, Biophysics Institute, Universidade Federal do Rio de Janeiro, Rio de Janeiro, Brazil

**Keywords:** Positive Affect, PANAS, emotion, working memory, event-related potential (ERP), contralateral delay activity (CDA), individual variability

## Abstract

Some studies have demonstrated a beneficial role of Positive Affect on working memory (WM) by either applying protocols of mood induction or assessing naturally occurring state Positive Affect. However, there are no studies directly linking Positive Affect as a stable personality-like trait with WM. We aimed to address this potential relationship using the Positive and Negative Affect Schedule scale and contra-lateral delay activity (CDA) as measures of trait Positive Affect and WM Capacity, respectively. We also sought to investigate the impact of a neutral or unpleasant emotional state on this relationship. Participants performed a change detection task, while a neutral or an unpleasant emotional state was induced. Our results showed a positive robust correlation between trait Positive Affect and WM Capacity for both neutral and unpleasant emotional states, as revealed by the neuroelectrophysiological gold-standard measure of WM, namely, CDA. These data suggest a tangible role of trait Positive Affect in the cognitive ability of maintaining goal-relevant information in WM, such that even a highly disruptive state is not sufficient to corrupt this relationship.

## Introduction

Positive emotions can lead to feeling more active and enthusiastic, and they have been associated with psychological health (Vazquez, 2017) and well-being (Burton and King, 2009). The amount and the tendency to experience positive emotions are together known as Positive Affect, which can be described from the perspective of either a transient mood (state) or a stable personality (trait) (Watson et al., 1988). When referring to trait, Positive Affect is associated with more frequent and intense episodes of pleasant state (Watson and Tellegen, 1985; Watson, 2002). It has been suggested that Positive Affect influences cognitive processes by increasing cognitive flexibility and/or by fostering goal-pursuit motivation (Gray, 2001; Isen and Reeve, 2005; Nadler et al., 2010).

Working memory (WM) is a cognitive system with limited capacity responsible for keeping goal-relevant information in focus (Goldman-Rakic, 1996; Cowan, 2001; Baddeley, 2012). WM allows the manipulation, use, and recall of relevant information and, if necessary, enables behavior changes to better cope with the challenge of achieving a goal (D’Ardenne et al., 2012; Stout et al., 2013). Some studies have investigated whether a direct effect of state Positive Affect on WM exists (Carpenter et al., 2013; Yang et al., 2013; Brose et al., 2014; Storbeck and Maswood, 2016). For example, both Yang et al. (2013) and Storbeck and Maswood (2016) observed better performance on a WM task by individuals in whom a positive mood had been induced. These studies focused on the affect state by applying different protocols of mood induction (Yang et al., 2013; Storbeck and Maswood, 2016) or assessing naturally occurring state Positive Affect (Brose et al., 2014). Affect trait and state may influence cognition in a different and interactive way, and considering affect trait is important (Dunn et al., 2010; Harlé and Sanfey, 2010; Hur et al., 2015; Riepl et al., 2016). Furthermore, no neuroelectrophysiological studies have addressed this association.

The contralateral delay activity (CDA) is a robust neuroelectrophysiological biomarker for WM Capacity (Vogel and Machizawa, 2004; Luria et al., 2016). An advantage of CDA in relation to other behavioral/neural measures of WM capacity is that it allows us to isolate in time the sustained activity exclusively related to the maintenance of items in WM (Fukuda et al., 2010). This event-related potential (ERP) is a great representative of the limited capacity of WM, as the amplitude of CDA increases along with the number of items to be maintained in WM but reaches a limit at approximately four items (Vogel and Machizawa, 2004; Vogel et al., 2005).

Previously, using measures of CDA, we showed that an unpleasant emotional state diminishes the WM capacity limit, demonstrating the disruptive effect of unpleasant emotional stimuli on WM (Figueira et al., 2017). Indeed, unpleasant stimuli and states can blunt goal-directed behavior and impact our decisions (Vuilleumier and Schwartz, 2001; Erthal et al., 2005; Dolcos and McCarthy, 2006; Pereira et al., 2006, 2010; Fernandes et al., 2013; Stout et al., 2013). In this vein, Sanchez et al. (2015) observed that neural reactivity for distractive unpleasant images is attenuated by trait Positive Affect, suggesting a potential crossplay between trait Positive Affect and the processing of unpleasant stimuli. In fact, Hur et al. (2015) supports the idea that trait Positive Affect may play a protective role as a compensatory mechanism in cognitive control when the individual is experiencing an unpleasant state.

To our knowledge, there is a gap in the literature regarding the influence of trait Positive Affect on WM using a neuroelectrophysiological approach. The current study aimed to fill this gap using the CDA, a gold-standard neuroelectrophysiological index of WM, to elucidate the putative interplay between trait Positive Affect and WM Capacity. Additionally, we investigated whether the induction of an unpleasant emotional state would disrupt this relationship.

## Materials and Methods

### Participants

The sample consisted of 33 participants (undergraduate students) from Figueira et al. (2017) and three added participants, for a total of 36 participants. The data sets of seven participants had to be excluded due to excessive behavioral errors (2), the production of extremely noisy data (2) and extensive eye movements (3). The remaining 29 participants (19 women) had a mean age of 21.67 years (*SD* = 4.69). All the participants were right-handed (Oldfield, 1971), reported normal color vision and normal or corrected-to-normal visual acuity. They also reported no psychiatric or neurological problems and were not using any central nervous system drugs.

### Stimuli and Procedure

The emotional state was created by the presentation of 120 pictures (20° × 16°) that were equally distributed in two categories: neutral (intact bodies) and unpleasant (mutilated bodies). They were presented in a blocked fashion to ensure the induction of a sustained modulatory effect by emotional picture viewing, as tested in Figueira et al. (2017). The neutral and unpleasant picture categories differed in both valence and arousal (see Supplementary Material, item 1).

**FIGURE 1:**
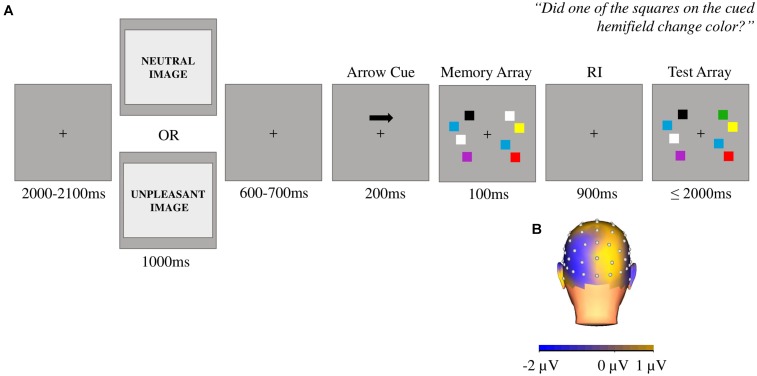
**(A)** Example of the sequential order of events in a trial. Each trial began with a fixation cross that remained on the screen throughout the entire trial. After 2000 to 2100 ms from the fixation cross onset, a neutral or an unpleasant picture was presented for 1000 ms in a blocked fashion. The change detection task started at 600 to 700 ms following the offset of the picture with an arrow cue for 200 ms. The arrow cue was replaced with a 100 ms memory array. The arrow pointed to the left on half of the trials. Then, there was a 900 ms retention interval (RI), followed by a test array. Participants were instructed to respond as quickly as possible and to try not to commit errors. **(B)** Topographical map showing the expected contralateral negativity during the RI arising from the left parietal-occipital electrodes when the cued hemifield was on the right.

After picture offset, the participants performed a change detection task (Vogel and Machizawa, 2004) that consisted of an arrow cue pointing to the to-be-attended hemifield and two sequential arrays (the memory array and the test array) of 2 or 4 colored squares in both hemifields (**Figure 1A**). The participants were instructed to covertly shift their attention toward the cued hemifield and press one of two buttons to indicate whether one of the squares changed color in the test array in relation to the previous memory array. The memory and test arrays differed in 50% of the trials. The uncued hemifield remained unchanged for all trials. For more information regarding the experimental paradigm, see Figueira et al. (2017).

### Trait Positive Affect Evaluation

Individual trait Positive Affect was assessed with the Positive and Negative Affect Schedule (PANAS) (Watson et al., 1988) at the beginning of the experimental session. This scale comprises two subscales consisting of 10 adjectives each: Positive Affect and Negative Affect. Because the aim of this study was to evaluate trait Positive Affect, only the Positive Affect dimension score was considered. To reflect TRAIT-LIKE Positive Affect stability, we instructed the participants to report the amount of positive emotions they experienced in general (Watson et al., 1988).

### Electroencephalogram (EEG) Recording

The EEG data were recorded and analyzed using our standard procedures (Figueira et al., 2017). As the CDA reflects visual WM processing (Luria et al., 2016), we computed CDA waveforms from the parietal-occipital P3/P4 and O1/O2 electrode pairs (Vogel and Machizawa, 2004) within an epoch of 1200 ms, starting 200 ms prior to the onset of the arrow cue and ending 1000 ms later. The mean peak amplitude was obtained over a 500–1000 ms time window during the retention interval (RI), in which the contralateral negativity of CDA is observed (**Figure 1B**).

### Statistical Analysis

To ensure that the results reported in Figueira et al. (2017) were not affected by the added participants, we replicated the following analysis: the mean peak amplitude obtained over the CDA time window was submitted to a repeated-measures ANOVA with the within-subject factors *site* (P3/P4 vs. O1/O2), *number of squares* (2 vs. 4) and *emotional state* (neutral vs. unpleasant). The Newman–Keuls procedure was used to test for *post hoc* differences when applicable.

To investigate the relationship between trait Positive Affect and WM Capacity, we conducted a robust Spearman correlation analysis (Rousselet and Pernet, 2012) between the PANAS Positive Affect score and the increase in CDA amplitude between the 2- and 4-squares task conditions during the unpleasant and neutral emotional states. The 95% confidence interval (CI) was computed using the MATLAB toolbox to perform robust correlation analysis available at http://sourceforge.net/projects/robustcorrtool/ (Pernet et al., 2013), based on bootstrapping procedures. Correlations were considered significant when CI did not encompass zero. Because CDA has a negative amplitude, for better visualization purposes, we multiplied the values by (-1). Thus, larger values represent a higher WM Capacity because they represent the increase in CDA amplitude between the 2- and 4-squares conditions.

## Results

The repeated-measures ANOVA revealed a significant main effect of *number of squares*, *F*(1,28) = 7.05; *p* < 0.05; however, the main effects of *site* and *emotional state* did not approach significance, *F*(1,28) = 1.79; *p* = 0.21 and *F*(1,28) = 0.38; *p* = 0.54, respectively. The interaction effect between the *number of squares* and *emotional state* was significant, *F*(1,28) = 5.66; *p* < 0.05. As demonstrated by *post hoc* analysis, the expected increment in CDA amplitude from 2 to 4 squares was revealed in the neutral emotional state, *p* < 0.05. On the other hand, the unpleasant emotional state affected the increment in CDA amplitude from 2 to 4 squares, *p* = 0.49. CDA’s amplitudes for neutral and unpleasant emotional states did not differ during the 2-squares condition, *p* = 0.25. During the 4-squares condition, the CDA amplitude was greater during the neutral emotional state than during the unpleasant emotional state, *p* < 0.05. The error results and CDA grand average waveforms can be found in the Supplementary Material, items 2 and 3.

**FIGURE 2:**
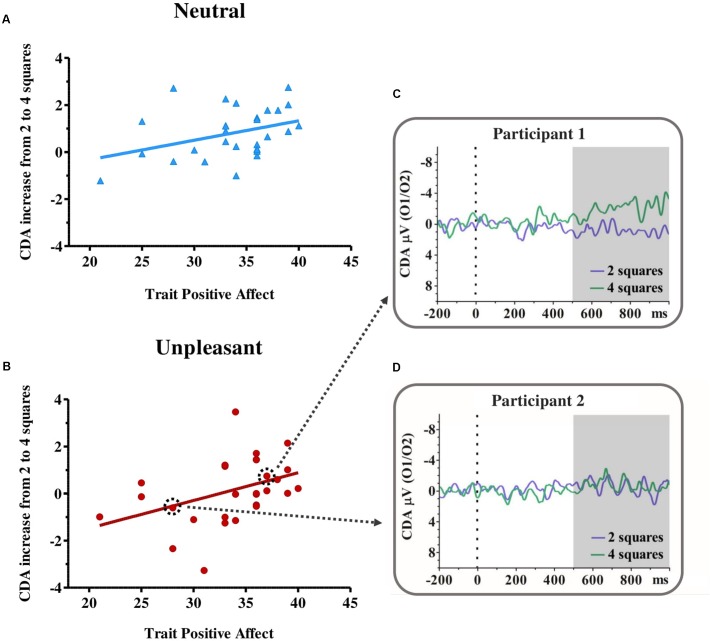
Correlation between participants’ working memory capacity [CDA mean amplitude (μV) increase from 2 to 4 colored squares] and trait Positive Affect during the neutral **(A)** and unpleasant emotional state **(B)**. On the right, two examples of the CDA waveform for the 2- (purple) and 4- (green) squares condition during the unpleasant emotional state are shown, obtained from an individual who had a higher score on trait Positive Affect [Participant 1 **(C)**] and an individual who had a low score on trait Positive Affect [Participant 2 **(D)**]. Note that in the correlations, there are overlapping points, 4 in the neutral state and 5 in the unpleasant state.

The mean Positive Affect score was *M* = 33.65 (*SD =* 4.64), whereas the mean WM Capacity, indexed by the increase in CDA amplitude from the 2- to 4-squares conditions (Vogel and Machizawa, 2004; Figueira et al., 2017), was *M* = 0.81 μV (*SD* = 1.04) for the neutral emotional state and *M* = 0.14 μV (*SD* = 1.37) for the unpleasant emotional state. The increase in CDA amplitude from 2 to 4 squares showed a significant positive robust correlation with Positive Affect score for the neutral emotional state (ρ = 0.41; *p* < 0.05, CI = [-0.706304 to 0.00408713]) and for the unpleasant emotional state (ρ = 0.49; *p* < 0.05, CI = [-0.764525 to 0.131]) (**Figure 2**). Hence, as the Positive Affect scores increase, the difference between CDA amplitude during the 2- and 4-squares conditions also increases, independently of whether the present emotional state is neutral or unpleasant.

## Discussion

In this work, we provide strong evidence for the beneficial effect of Positive Affect on WM Capacity. To our knowledge, this is the first study to provide evidence of a constructive relationship between Positive Affect and WM Capacity using a gold-standard neuroelectrophysiological marker. Our data indicate that trait Positive Affect increases with WM Capacity, even in the face of an unpleasant state.

Most research on the relationship between Positive Affect and cognition has focused on state Positive Affect. For example, in the systematic review performed by Vanlessen et al. (2016), the authors proposed that the transient experience of positive emotions grants a flexible management of attentional resources that are built on cognitive demand. However, the mechanism through which trait Positive Affect influences cognitive processes, such as WM, remains unclear. Therefore, our study addresses this topic and demonstrates a positive relationship between trait Positive Affect and WM Capacity, suggesting that trait Positive Affect enhances the cognitive ability of holding information in memory.

The literature greatly supports the idea that unpleasant emotional states or stimuli are highly disruptive to cognitive resources (Vuilleumier and Schwartz, 2001; Erthal et al., 2005; Dolcos and McCarthy, 2006; Pereira et al., 2006, 2010; Fernandes et al., 2013; Stout et al., 2013). Indeed, Figueira et al. (2017) have demonstrated that WM Capacity is diminished by unpleasant states, leading to a failure that may disrupt the ability to perform many activities on a daily basis. Surprisingly, our results demonstrate a positive correlation between trait Positive Affect and WM Capacity that is preserved even during a concurrent disruptive unpleasant state. This result may also be explained by a down-regulation of unpleasant emotions through emotion regulation, as the latter is positively related to both Positive Affect (Fredrickson, 2001) and WM (Xiu et al., 2016). Considering the absence of a pleasant-state condition in our study, we are unable to uncover the cognitive relationship between trait Positive Affect and emotional state as a whole. Addressing this issue in the future would be interesting.

Our study adds new data showing that WM Capacity is case sensitive to trait Positive Affect. Our results provide a new avenue toward understanding the interplay between Positive Affect and WM. In view of our results, we hypothesize that Positive Affect enhances WM Capacity through two distinct mechanisms: by assuming a mediator role by maintaining a goal throughout a task and by shielding task disruption from unpleasant distractors. Further studies should address how trait Positive Affect can enable better coping strategies in the face of unpleasant emotional states or stimuli.

## Ethics Statement

This study was carried out in accordance with the recommendations of Federal Fluminense University, University Hospital Ethics Committee (HU). The protocol was approved by the University Hospital Ethics Committee (HU, CAAE: 53505615.0.0000.5243). All the subjects provided written informed consent in accordance with the Declaration of Helsinki.

## Author Contributions

ID, MP, LdO, and JF developed the study concept and the study design. JF and IL performed the data collection. JF and LP performed the data analysis and interpretation under the supervision of ID. JF and LP drafted the manuscript. ID, MP, LdO, and EV substantially contributed to the interpretation of the data and provided important critical revisions. All the authors approved the final version of the manuscript. They also agreed to be accountable for all aspects of the work.

## Conflict of Interest Statement

The authors declare that the research was conducted in the absence of any commercial or financial relationships that could be construed as a potential conflict of interest.
