# Supplementary material for: “Keep That in Mind!” The Role of Positive Affect in Working Memory for Maintaining Goal-Relevant Information
Source: Front Psychol. 2018 Jul 19;9:1228. doi: 10.3389/fpsyg.2018.01228 (PMC6060567; doi:10.3389/fpsyg.2018.01228)
Supplement: Supplementary file 1 [file Table_1.DOCX]

Supplementary Material

“Keep that in Mind!” The Role of Positive Affect in Working Memory for Maintaining Goal-Relevant Information.

**Jessica Sanches Braga Figueira^1^, Luiza Bonfim Pacheco^1^, Isabela Lobo^2^, Eliane Volchan^3^, Mirtes Garcia Pereira^1^, Leticia de Oliveira^1^, Isabel Antunes David^1^***

^1^ Laboratory of Behavioral Neurophysiology, Physiology and Pharmacology Department, Biomedical Institute, Universidade Federal Fluminense, Niterói, Brazil.

^2^ Laboratory of Psychobiology, NUPEM, Universidade Federal do Rio de Janeiro, Rio de Janeiro, Brazil.

^3^ Laboratory of Neurobiology II, Biophysics Institute, Universidade Federal do Rio de Janeiro, Rio de Janeiro, Brazil.

*** Correspondence:**Isabel A. David

# [isabeldavid@id.uff.br](mailto:isabeldavid@id.uff.br)

# Selected IAPS Images

Neutral: 2383; 2230; 2190; 2200; 2480; 2575; 2485; 2570; 2630; 2381; 2210; 2214; 2215; 2495; 2221; 2516.

Unpleasant: 3000; 3010; 3030; 3053; 3060; 3063; 3064; 3071; 3080; 3100; 3102; 3110; 3120; 3130; 3140; 3150; 3170; 3250; 3400; 3550.

The remaining 24 images were obtained from the worldwide web. Following the protocol developed by Lang and colleagues (Lang and Greenwald, 1988; Lang et al., 2008), all the images were previously assessed on a 1–9 scale in terms of valence (from negative to positive) and arousal (from low to high) by a different group of graduate students.

Overall, the neutral and unpleasant pictures differed significantly from each other with respect to both valence (M=5.06, SD=.42 and M=2.08, SD=.52, respectively, *t*(59)=35.06; *P*<0.01) and arousal (M=3.29, SD=0.48 and 6.89 SD=0.49, respectively, *t*(59)= –41.41; *P*<0.01) ratings.

# Supplementary Table

The total number of errors included anticipation (reaction time (RT)<150ms), slow responses (RT>2000ms), and incorrect key-press responses. These values were submitted to a repeated-measures analysis of variance with the number of squares (2 vs. 4 squares) and emotional state (neutral vs. unpleasant) as factors. As expected, there was a main effect of number of squares (F(1,28)=233.89, p<0.001), meaning that participants committed more errors during the 4-squares condition than during the 2-squares condition. The main effect of emotional state did not approach significance, F(1,28)=0.94, p=0.34. The interaction between the factors did not approach significance either, F(1,28)=0.32, p=0.58. The values for the mean and standard deviation are shown in the table below.

|  | Neutral | | Unpleasant | |
| --- | --- | --- | --- | --- |
| Number of Squares | 2 | 4 | 2 | 4 |
| Mean (SD) | 3.03 (2.78) | 14.62 (4.81) | 4.31 (6.32) | 15.31 (5.0) |

# Supplementary Figures


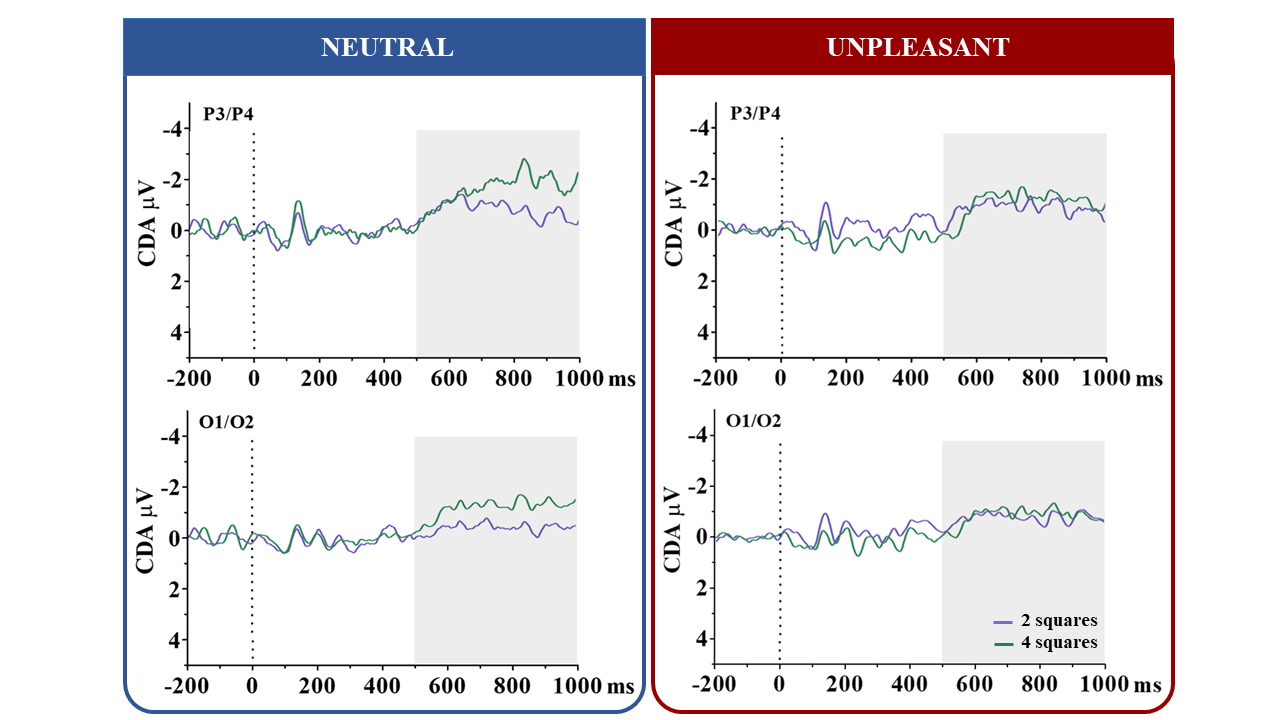


**Supplementary Figure 1.** Contralateral delay activity (CDA) grand average waveforms from 29 participants for parietal and occipital (P3/P4 and O1/O2) electrode pairs upon presentation of the change detection task during the neutral (left, blue contour) and unpleasant (right, red contour) emotional states. The figure depicts the increase in CDA amplitude from 2 (purple lines) to 4 (green lines) to-be-remembered items. The CDA was measured in the 500–1000ms time window after the onset of the arrow (gray shaded rectangle).
